# Evaluation of PET/CT imaging with [^89^Zr]Zr-DFO-girentuximab: a phase 1 clinical study in Japanese patients with renal cell carcinoma (Zirdac-JP)

**DOI:** 10.1093/jjco/hyae075

**Published:** 2024-06-12

**Authors:** Noboru Nakaigawa, Hisashi Hasumi, Daisuke Utsunomiya, Keisuke Yoshida, Yoshinobu Ishiwata, Takashi Oka, Colin Hayward, Kazuhide Makiyama

**Affiliations:** Department of Urology, Yokohama City University, Yokohama City, Kanagawa 236-0004, Japan; Department of Urology, Kanagawa Cancer Center, Yokohama City, Kanagawa 241-8515, Japan; Department of Urology, Yokohama City University, Yokohama City, Kanagawa 236-0004, Japan; Department of Diagnostic Radiology, Yokohama City University Graduate School of Medicine, Yokohama, Kanagawa 236-0004, Japan; Department of Diagnostic Radiology, Yokohama City University Graduate School of Medicine, Yokohama, Kanagawa 236-0004, Japan; Department of Radiology, Yuai Clinic Diagnostic Imaging, 1-6-2, Kita-shinyokohama, Kohoku-ku, Yokohama, Kanagawa 223-0059, Japan; Department of Diagnostic Radiology, Yokohama City University Graduate School of Medicine, Yokohama, Kanagawa 236-0004, Japan; Telix Pharmaceuticals Japan K.K., KRP #4 Building, 93 Chudoji-Awata-machi, Shimogyu-ku, Kyoto-shi, Kyoto, Japan; Telix Pharmaceuticals, 55 Flemington Road, North Melbourne, VIC 3051, Australia; Department of Urology, Yokohama City University, Yokohama City, Kanagawa 236-0004, Japan

**Keywords:** clear cell renal cell carcinoma, [89Zr]Zr-DFO-girentuximab PET/CT imaging, renal cancer

## Abstract

**Background:**

PET/CT imaging with Zirconium-89 labeled [^89^Zr]Zr-DFO-girentuximab, which targets tumor antigen CAIX, may aid in the differentiation and characterization of clear cell renal cell carcinomas (RCC) and other renal and extrarenal lesions, and has been studied in European and American cohorts. We report results from a phase I study that evaluated the safety profile, biodistribution, and dosimetry of [^89^Zr]Zr-DFO-girentuximab in Japanese patients with suspected RCC.

**Methods:**

Eligible adult patients received 37 MBq (± 10%; 10 mg mass dose) of intravenous [^89^Zr]Zr-DFO-girentuximab. Safety and tolerability profile was assessed based on adverse events, concomitant medications, physical examination, vital signs, hematology, serum chemistry, urinalysis, human anti-chimeric antibody measurement, and 12-lead electrocardiograms at predefined intervals. Biodistribution and normal organ and tumor dosimetry were evaluated with PET/CT images acquired at 0.5, 4, 24, 72 h and Day 5 ± 2 d after administration.

**Results:**

[^89^Zr]Zr-DFO-girentuximab was administered in six patients as per protocol. No treatment-emergent adverse events were reported. Dosimetry analysis showed that radioactivity was widely distributed in the body, and that the absorbed dose in healthy organs was highest in the liver (mean ± standard deviation) (1.365 ± 0.245 mGy/MBq), kidney (1.126 ± 0.190 mGy/MBq), heart wall (1.096 ± 0.232 mGy/MBq), and spleen (1.072 ± 0.466 mGy/MBq). The mean effective dose, adjusted by the radioactive dose administered, was 0.470 mSv/MBq. The radiation dose was highly accumulated in the targeted tumor, while any abnormal accumulation in other organs was not reported.

**Conclusions:**

This study demonstrates that [^89^Zr]Zr-DFO-girentuximab administered to Japanese patients with suspected RCC has a favorable safety profile and is well tolerated and has a similar dosimetry profile to previously studied populations.

## Teaser

**Trial registration:**  ClinicalTrials.gov, NCT04496089; JapicCTI-205 399. Registered 14 July 2020, https://www.clinicaltrials.gov/study/NCT04496089?id=NCT04496089&rank=1

## Introduction

Renal cell carcinoma (RCC) carries a substantial global burden accounting for 2.2% of the total cancer incidence with 431 288 new cases diagnosed and 179 368 deaths reported (1.8% total cancer deaths) in 2020 [1]. In Japan, more than 13 000 patients with kidney cancer were recorded in 2017, with over 7000 deaths with an estimated cost of 114 712 disability-adjusted life years [2].

Clear cell renal cell carcinoma (ccRCC) is the most common and aggressive histological subtype, comprising up to 90% of all RCCs and accounting for the majority of metastases and deaths [3–6]. Stage at diagnosis markedly impacts 5-year survival, declining to 12% for stage IV from 93% for stage I disease. Disease-specific survival is worse with ccRCC as it tends to be discovered at a more advanced stage [7]; thus, early and definitive diagnosis is important for improved patient outcomes. Current diagnostic tools include conventional imaging and biopsy, which have major limitations. Conventional imaging, such as computed tomography (CT), magnetic resonance imaging (MRI), and ultrasound, relies on structural imaging and has inadequate diagnostic performance to reliably detect, characterize, and differentiate ccRCC and other renal and extrarenal lesions. Renal biopsy is invasive, and feasibility depends on anatomic tumor location. It has a high nondiagnostic rate (up to 15%) and cannot provide information on tumor characteristics [8–10]. After renal biopsy, up to 20–25% of small renal masses (≤4 cm) are misclassified as malignant and thus unnecessarily involves surgical removal, with one study reporting a 10% rate of surgical complications in patients with benign lesions who underwent elective nephrectomy for suspected RCC [11,12]. Renal mass biopsy can lead to complications including hematoma (4.9%), clinically significant pain (1.2%), gross hematuria (1.0%), pneumothorax (0.6%), and hemorrhage (0.4%) [13]. In addition, it poses the risk of tumor seeding along the biopsy needle track [14].

The majority of all ccRCCs overexpress the antigen carbonic anhydrase IX (CAIX) on the surface of the tumor cells [15], suggesting the potential value of CAIX as a marker for identification of ccRCC. The monoclonal antibody, girentuximab, is known to target CAIX and recently, positron emission tomography (PET)/CT imaging using radiolabeled girentuximab has proven benefit in detecting CAIX expression in lesions that are indeterminate in standard PET/CT imaging [16–18].

Zirconium-89 has shown great potential as a versatile PET-imaging tool, particularly for radiolabeling monoclonal antibodies to assess antigen expression, analyze biodistribution, plan treatment, and evaluate response to anticancer therapies [19]. [^89^Zr]Zr-DFO-girentuximab has shown value in solving diagnostic dilemmas in ccRCC [18] and in increasing the detection rate compared with CT alone in newly diagnosed metastatic disease [20]. We report results from a phase I study that evaluated the safety profile, biodistribution, and dosimetry of [^89^Zr]Zr-DFO-girentuximab in Japanese patients with suspected RCC.

## Patients and methods

### Study design

This was a single-center, open-label, prospective phase I (ClinicalTrials.gov identifier NCT04496089; JapicCTI-205 399). The primary objective was to assess the safety and tolerability of [^89^Zr]Zr-DFO-girentuximab in Japanese patients with suspected RCC (including ccRCC). Secondary objectives were to determine the whole-body radiation dosimetry of [^89^Zr]Zr-DFO-girentuximab, compare image quality by imaging condition, and assess tumor dosimetry by assessment of biodistribution/tumor uptake.

### Patients

Adult patients (≥20 years of age) with clinical suspicion of RCC (CT or MRI indicating renal mass) and sufficient life expectancy to participate in the study were enrolled. Patients agreed to use barrier contraceptives during the study period and for at least 42 (females) or 90 d (males, who also agreed to refrain from sperm donation) after injection of [^89^Zr]Zr-DFO-girentuximab. Exclusion criteria included hypersensitivity to girentuximab or desferrioxamine (DFO); metastatic renal tumor; treatment requirement for other active malignant diseases during study participation; chemotherapy, radiotherapy, and immunotherapy within 4 weeks prior to injection of [^89^Zr]Zr-DFO-girentuximab or persistent grade 1 (per National Cancer Institute Common Terminology Criteria for version 5.0 [NCI-CTCAE v5.0]) adverse events (AEs) due to such therapy; scheduled treatment with anticancer agents during time between injection of [^89^Zr]Zr-DFO-girentuximab and imaging acquisition; exposure to murine or chimeric antibodies with the last 5 years; administration of any radionuclide within 10 half-lives of the radionuclide; serious but not life-threatening diseases, as per investigator’s discretion; pregnant or lactating females or females of childbearing potential who were suspected to be pregnant by blood test at screening within 24 h before injection of [^89^Zr]Zr-DFO-girentuximab or by same-day pre-imaging urine pregnancy test; administration of any drug for clinical research or study within 30 d administration of [^89^Zr]Zr-DFO-girentuximab consent alone; renal impairment with glomerular filtration rate of 45 mL/min/1.73 m^2^ or less; socially vulnerable; or determined by investigator to be ineligible for any other reason.

### [^89^Zr]Zr-DFO-girentuximab administration and PET/CT imaging

A single injection of 37 MBq (± 10%) of [^89^Zr]Zr-DFO-girentuximab containing 10 mg of girentuximab was administered intravenously over approximately 3 min. Patients were monitored for 30 min following administration. The syringe was flushed once using 15 mL NaCl 0.9% post-administration, and the intravenous line and syringe were measured for residual activity.

Whole body PET/CT imaging (from the base of skull to the thigh) was performed with a Toshiba Celestion PET/CT scanner in the supine position at 0.5, 4, 24, and 72 h, and Day 5 ± 2 after administration using non-contrast-enhanced and low-dose CT for attenuation correction. Images in both Time of Flight (TOF) and conventional PET were acquired at 5, 10, 15, and 20 min after [^89^Zr]Zr-DFO-girentuximab administration. Imaging diagnosis/assessment of the tumor was made with cross-sectional and qualitative review of all acquisition conditions (images acquired by reconstruction with TOF or non-TOF/conventional PET). Image evaluators had specialized knowledge and experience in image interpretation and clinical diagnosis in the field and were trained on the image evaluation method for this study. The proportion of excellent or good images were evaluated by acquisition condition on 4 scales (excellent, good, fair, and unevaluable) for assessment of image quality. Evaluators provided image interpretation independently and confirmed results by a blinded central imaging assessment committee consisting of three trained readers.

### Safety assessment

Safety and tolerability assessment was based on physical examination, vital signs, hematology, serum chemistry, urinalysis, and 12-lead electrocardiograms (ECGs) at predefined time intervals (screening, pre-administration, Day 1 (24 h after administration), Day 3 (72 h after administration), Day 5 ± 2 d (120 h after administration), and 7 d after Day 5 ± 2 d. Treatment-emergent adverse events (TEAEs) reporting period was from the start of the [^89^Zr]Zr-DFO-girentuximab injection up to 42 d after administration. AEs were graded according to NCI-CTCAE v5.0). Blood sampling for human anti-chimeric antibody (HACA) measurement was performed at screening and on the day of last visit (7 d after Day 5 ± 2 d).

### Dosimetry and imaging quality

The effective dose (mSv/MBq) in the whole body and the absorbed dose (mGy/MBq) in each organ were evaluated. To set volumes of interest (VOI), kidneys (left and right), liver, spleen, heart content (not including ascending and descending aorta), and lumbar vertebrae 1–5 (as surrogate for red marrow) were manually drawn and then segmented using either the PET image or corresponding CT image. VOI of all segmented organs were then copied onto all other time points to calculate the time activity curves for all organs. The anatomical tumor volumes for use in tumor dosimetry were segmented on the pre-study diagnostic contrast-enhanced CT. Absorbed and effective dose calculations were performed using OLINDA. Organ masses were not adapted to individual subject organ masses. The absorbed doses to the tumors were determined using the spherical model [21]. For the specific tumor masses, the dose was calculated in QDOSE by interpolation using a power function to the volume/mass assuming a density of 1.06 g/cm^3^.

Tumor uptake was evaluated based on [^89^Zr]Zr-DFO-girentuximab accumulation in tumors or in surrounding regions according to the PET/CT images acquired on Day 3 and Day 5 ± 2 d and compared with the reference images of CT or MRI performed prior to enrollment. Dose distribution by tissue and whole-body was determined considering individual anatomical data and by phantom data based on medical internal radiation dose method using OLINDA.

### Statistical analysis

Pharmacokinetic and pharmacodynamic results were analyzed by standardized non-compartmental methods with Phoenix WinNonlin Version 8.3. The following parameters were calculated on whole blood and plasma radioactivity as appropriate using elapsed time from dosing: area under the concentration-time curve to last time point (AUC_0-t_), area under the concentration-time curve extrapolated to infinity (AUC_0-∞_), maximum serum concentration (C_max_), time to C_max_ (T_max_), estimate of the terminal elimination rate constant (λ_z_), and half-life (t_½_). A minimum of three quantifiable data points were required for calculation. Samples for pharmacokinetic analysis were collected pre-injection on Day 0 and 0.5, 1, 2, 4, 24, 72, and 120 h following injection. All primary and secondary variables were described using exploratory data analysis.

## Results

A total of six patients were enrolled, and six (four males and two females) were eligible and received a single intravenous administration of [^89^Zr]Zr-DFO-girentuximab. One patient did not meet eligibility criteria due to a glomerular filtration range that met exclusion criteria. Demographics and baseline characteristics are provided in Table 1. The median age was 67.5 years (mean ± SD: 62.8 ± 14.19), and the median body surface area was 23.97 kg/m^2^ (24.4 ± 13.07).

**Table 1:** Demographics and baseline characteristics.

**Characteristics**	***N* = 6**
Age (y) Mean ± SD Median Min, max	62.8 ± 14.19 67.5 38, 77
Sex [*n* (%)] Male Female	4 (66.7) 2 (33.3)
Race [*n* (%)] Asian	6 (100.0)
Weight (kg) Mean ± SD Median Min, max	64.8 ± 11.50 65.9 48.7, 81.0
Height (cm) Mean ± SD Median Min, max	162.7 ± 9.78 162.1 150.7, 178.0
BMI (kg/m^2^) Mean ± SD Median Min, max	24.4 ± 3.07 23.97 20.1, 28.8

BMI, body mass index; Max, maximum; Min, minimum; SD, standard deviation.

Age (y) at date of informed consent

### Safety

No TEAEs were reported. There were no clinically significant abnormalities in laboratory parameters (hematology, coagulation, urinalysis), vital signs (blood pressure, pulse rate, body temperature), or ECG. Vital signs and blood test results are shown in Supplemental Table 1. HACA was not detected.

### Whole-body distribution and dose estimation

Results of dosimetry after a single intravenous administration of [^89^Zr]Zr-DFO-girentuximab showed that radioactivity was widely distributed in the body, and that the normalized absorbed dose, calculated as the ratio of absorbed dose and the radioactive dose administered, was highest in the liver, kidney, heart wall, and spleen (Table 2).

**Table 2:** Biodistribution of radioactivity (normalized absorbed dose mGy/MBq)^a^.

**Target organ**	***N* = 6** **Mean ± SD**
Adrenals	0.842 ± 0.119
Brain	0.285 ± 0.040
Breasts (*n* = 2)	0.369 ± − ^b^
Esophagus	0.526 ± 0.088
Eyes	0.286 ± 0.0413
Gallbladder wall	0.751 ± 0.0666
Left colon	0.519 ± 0.0921
Small intestines	0.468 ± 0.0397
Stomach wall	0.530 ± 0.0594
Right colon	0.512 ± 0.0425
Rectum	0.429 ± 0.0533
Heart wall	1.096 ± 0.232
Kidneys	1.126 ± 0.190
Liver	1.365 ± 0.245
Lungs	0.470 ± 0.0724
Osteogenic cells	0.550 ± 0.0534
Ovaries (*n* = 2)	0.506 ± − ^b^
Pancreas	0.613 ± 0.102
Prostate (*n* = 4)	0.371 ± 0.0204
Salivary glands	0.329 ± 0.0310
Red marrow	0.700 ± 0.0934
Spleen	1.072 ± 0.466
Testes (*n* = 4)	0.281 ± 0.0161
Thymus	0.502 ± 0.0668
Thyroid	0.356 ± 0.0350
Urinary bladder wall	0.361 ± 0.0171
Uterus (*n* = 2)	0.484 ± − ^b^

SD, standard deviation.

^a^Absorbed dose (mGy) was divided by the actual radioactive dose administered (MBq).

^b^n = 2, SD is not represented.

The mean effective dose adjusted by the radioactive dose administered was 0.470 mSv/MBq. Tumor dose measurement was evaluated from the radioactive dose in the tumor of the kidney and the presence or absence of abnormal accumulation of radioactive doses in other organs. The absorbed dose in the kidney tumor ranged from 0.669 to 6.549 mGy/MBq, and abnormal accumulation in other organs was not reported in any patients (Table 3).

**Table 3:** Pharmacodynamics − radiation dose of tumor in kidney by patient.

**Sex/age**	**Lesion location**	**Normalized absorbed dose** ^**a**^ **(mGy/MBq)**
M/67	Right kidney	1.578
F/55	Left kidney	0.660
F/38	Left kidney	2.590
M/72	Left kidney	6.161
M/68	Left kidney	6.549
M/77	Right kidney	2.265

M, male. F, female. Age (y) at date of study medication administration

^a^Absorbed dose (mGy) was divided by the actual radioactive dose administered (MBq).

### Image quality assessment by acquisition condition

**Figure 1:**
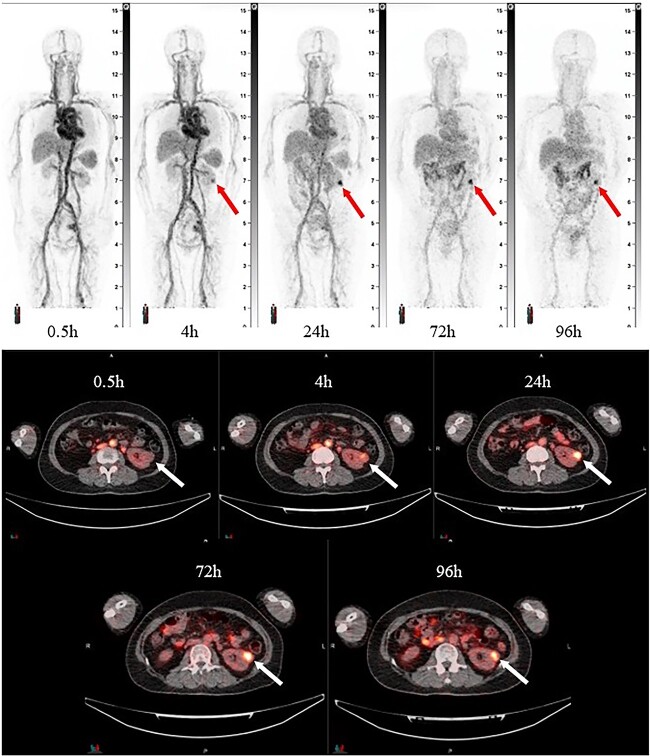
Representative [89Zr]Zr-DFO-girentuximab PET/CT images at 0.5, 4, 24, 72, and 96 h following injection. Top: maximum intensity projection images. Bottom: axial PET/CT fusion images. Arrows indicate the location of left renal lesion.

 Image quality gradually improved over time after injection to the last time point at 20 min. Image quality was judged as Excellent or Good in images acquired 15 min after injection in all patients and thereafter in both TOF (+) and TOF (−). Images acquired 20 min after injection in TOF (+) were judged as Excellent in five patients (83.3%). Representative PET images are in Fig. 1.

### Pharmacokinetics

Whole blood and serum samples were available for four and two patients, respectively. Two patients were excluded from calculation of descriptive statistics of PK parameters related to λz because the observation period for calculation of λz was less than two times of the estimated t_1/2_, and AUC_0-t_ was less than 80% of AUC_0-∞_. Four patients were excluded from calculation of descriptive statistics of PK parameters related to λβ because the observation period for calculation of λz was less than two times of the estimated t_1/2_. Therefore, arithmetic mean and SD could not be calculated because of *n* = 2, and minimum and maximum are shown.

**Figure 2:**
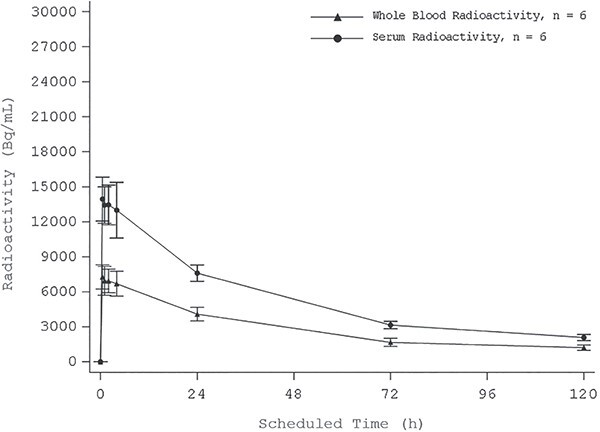
Mean whole blood and serum radioactivity–time profile (linear).

The median times (T_max_) for whole blood and serum radioactivity to reach the maximum concentration (C_max_) were 0.6 and 1.5 h after dosing and concentrations declined slowly thereafter (Fig. 2). Whole blood and serum radioactivity were detected in all patients at 96 h after dosing. Whole blood radioactivity attained C_max_ within 0.55–1.02 h (median T_max_: 0.570 h). Serum radioactivity attained C_max_ after 0.57–4.02 h (median T_max_: 1.51 h) and was slower than whole blood. The mean ± SD of elimination half-life of whole blood was 37.8 h ±2.01, and this value was close to that of serum obtained from two patients (34.3 and 37.2 h). The mean C_max_ ± SD was 7280 Bq/mL ± 1042 in whole blood and 14 400 Bq/mL ± 2265 in serum. The mean area under the concentration-time curve truncated at 96 h (AUC0-96) was 302 000 Bq h/mL ± 39 460 and 553 000 Bq h/mL ± 38 640 in whole blood and serum, respectively. The mean total body clearance and volume of distribution in whole blood were 0.101 L/h and 5.48 L, respectively, and these values were about twice as high than those calculated from serum in two patients, suggesting a low distribution of radioactivity into red blood cells.

## Discussion

An unmet need exists for a tool that provides accurate and noninvasive characterization and differentiation of ccRCC. Among Asian American patients with ccRCC, Japanese patients are the oldest at diagnosis and have the lowest overall survival [22]. This study is the first to demonstrate the favorable safety profile and tolerability of [^89^Zr]Zr-DFO-girentuximab in Japanese patients with suspected RCC. No TEAEs, clinically meaningful changes in laboratory parameters or vital signs, physical examination findings, and other safety-related observations were reported. HACA was not detected in any patient.

[^18^F]-fluorodeoxyglucose is currently the dominant radiotracer used for renal cancer imaging; however, its low sensitivity renders it unsuitable for staging patients with RCC [23]. Immuno-PET combines the sensitivity of PET with the specificity of antibodies. ^124^I-labeled -girentuximab has been shown to be highly accurate and specific for ccRCC [24]. ^89^Zr is an emerging radionuclide with several advantages compared with ^124^I, including superior spatial resolution and a longer period by which it can internalize and residualize after binding to cell surfaces. ^89^Zr-labeled girentuximab has up to 3-fold higher tumor uptake than ^124^I-labeled girentuximab [23].

The whole-body and organ-specific radiation dosimetry of [^89^Zr]Zr-DFO-girentuximab is comparable to a previous study [25]. The radiation dose was highly accumulated in the targeted tumor, and no abnormal accumulation was found in organs. The tumor dose uptake range (0.7–6.6 mGy/MBq) overlapped with the range (1.90–11.6 mGy/MBq) reported in a previous study with a similar radioactive dose [25]. While not reported by Merckx et al. [25], given the study was conducted in Europe, it is likely that the sample was not homogenously Japanese patients, suggesting similar dosimetry results in Japanese and European populations. A limitation of this study includes small sample size; however, results are consistent with a phase 3 study of [^89^Zr]Zr-DFO-girentuximab PET/CT imaging for detection and characterization of ccRCC that included 284 patients in the study analysis.

## Conclusions

In summary, this study demonstrates that [^89^Zr]Zr-DFO-girentuximab has a favorable safety profile in Japanese patients and dosimetry profile that is in line with the literature. Results support existing data of the promising efficacy and clinical utility across varying populations of [^89^Zr]Zr-DFO-girentuximab PET/CT imaging for differentiation and characterization of ccRCCs.

## Abbreviations

***AE:*** adverse event

***AUC***_***0-t***_ ***:*** area under the concentration curve, to the final measured time point. 

***AUC***_***0-∞***_ ***:*** area under the concentration curve, extrapolated to infinity. 

***AUC***_***0–96***_ ***:*** area under the concentration curve, truncated at 96 h.

***CAIX:*** carbonic anhydrase IX.

***ccRCC*:** clear cell renal cell carcinoma.

***C***_***max:***_ maximum concentration.

***CT:*** computed tomography.

**DFO**: desferrioxamine.

***ECG:*** electrocardiogram.

***MIRD:*** medical internal radiation dose.

***NCI-CTCAE v5.0:*** National Cancer Institute Common Terminology Criteria for Adverse Events version 5.0.

***PET:*** positron emission tomography.

***RCC:*** renal cell carcinoma.

***SD:*** standard deviation.

***TEAE:*** treatment-emergent adverse event.

***T***_***max***_ ***:*** time to maximum concentration.

***T***_***1/2***_ ***:*** half-life. 

***TOF:*** time of flight.

***[*** ^***89***^ ***Zr]Zr-DFO-girentuximab:*** zirconium-89-labeled desferrioxamine-girentuximab.

***VOI:*** volume of interest 

***λz:*** estimate of the terminal elimination rate constant.

## Supplementary Material

Supplemental-table-vitals-heme-tests_hyae075

## Data Availability

The datasets used and/or analyzed during the current study are available from the corresponding author on reasonable request.
