# Supplementary material for: Evaluation of PET/CT imaging with [89Zr]Zr-DFO-girentuximab: a phase 1 clinical study in Japanese patients with renal cell carcinoma (Zirdac-JP)
Source: Jpn J Clin Oncol. 2024 Jun 12;54(8):873–9. doi: 10.1093/jjco/hyae075 (PMC11322881; doi:10.1093/jjco/hyae075)
Supplement: Supplemental-table-vitals-heme-tests_hyae075 [file supplemental-table-vitals-heme-tests_hyae075.docx]

**Supplemental Table 1. Results of chemistry, hematology, and urinalysis tests and vital signs of patients at baseline, 4, 24, 72, 120 hours following injection, and last visit.**

|  |  | **Time (hours) following injection of ^68^Ga-PSMA-11** | | | |  |
| --- | --- | --- | --- | --- | --- | --- |
|  | **Baseline**  **Mean ± SD** | **4**  **Mean ± SD** | **24**  **Mean ± SD** | **72**  **Mean ± SD** | **120**  **Mean ± SD** | **Last visit**  **Mean ± SD** |
| Chemistry |  |  |  |  |  |  |
| Alanine aminotransferase (uKat/L) | 0.439 ± 0.379 | 0.442 ± 0.402 | 0.439 ± 0.434 | 0.453 ± 0.394 | 0.389 ± 0.272 | 0.391 ± 0.199 |
| Albumin (g/L) | 41.667 ± 3.266 | 41.333 ± 2.805 | 40.500 ± 2.168 | 41.833 ± 3.061 | 40.833 ± 2.787 | 44.333 ± 2.503 |
| Alkaline phosphatase (uKat/L) | 1.624 ± 1.334 | 1.059 ± 0.568 | 1.063 ± 0.547 | 1.092 ± 0.469 | 1.054 ± 0.503 | 1.244 ± 0.515 |
| Amylase (uKat/L) | 1.267 ± 0.516 | 1.247 ± 0.527 | 1.281 ± 0.487 | 1.278 ± 0.472 | 1.242 ± 0.435 | 1.334 ± 0.528 |
| Aspartate aminotransferase (uKat/L) | 0.570 ± 0.577 | 0.564 ± 0.616 | 0.528 ± 0.544 | 0.478 ± 0.311 | 0.420 ± 0.222 | 0.428 ± 0.180 |
| Bilirubin (umol/L) | 14.250 ± 2.574 | 12.540 ± 3.849 | 13.395 ± 4.641 | 11.685 ± 6.062 | 10.830 ± 3.998 | 12.540 ± 3.694 |
| Calcium (mmol/L) | 2.329 ± 0.064 | 2.287 ± 0.041 | 2.267 ± 0.076 | 2.275 ± 0.0578 | 2.283 ± 0.038 | 2.370 ± 0.045 |
| Chloride (mmol/L) | 103.667 ± 1.967 | 104.000 ± 3.033 | 104.000 ± 2.192 | 104.332 ± 2.805 | 105.500 ± 2.429 | 103.500 ± 3.271 |
| Creatinine (umol/L) | 67.037 ± 14.335 | 64.385 ± 14.487 | 64.385 ± 13.654 | 66.005 ± 14.228 | 64.679 ± 13.597 | 64.974 ± 14.544 |
| Gamma glutamyl transferase (uKat/L) | 0.767 ± 1.088 | 0.736 ± 1.054 | 0.714 ± 0.983 | 0.717 ± 0.998 | 0.708 ± 0.986 | 0.730 ± 0.975 |
| Glucose (mmol/L) | 9.213 ± 3.622 | 8.214 ± 2.568 | 9.583 ± 2.809 | 6.808 ± 2.167 | 6.355 ± 1.758 | 7.474 ± 2.252 |
| Lipase (uKat/L) | 0.497 ± 0.129 | 0.486 ± 0.124 | 0.545 ± 0.188 | 0.550 ± 0.172 | 0.567 ± 0.143 | 0.545 ± 0.144 |
| Potassium (mmol/L) | 4.033 ± 0.333 | 3.800 ± 0.141 | 3.733 ± 0.151 | 4.083 ± 0.271 | 4.033 ± 0.186 | 4.133 ± 0.308 |
| Protein (g/L) | 69.000 ± 3.286 | 66.833 ± 3.488 | 67.000 ± 2.683 | 67.500 ± 4.087 | 67.167 ± 2.858 | 72.500 ± 3.728 |
| Sodium (mmol/L) | 140.333 ± 1.506 | 140.333 ± 2.944 | 140.667 ± 1.211 | 140.667 ± 2.658 | 141.500 ± 1.378 | 141.333 ± 2.160 |
| Urate (umol/L) | 318.218 ± 65.563 | 306.322 ± 57.760 | 319.209 ± 79.594 | 318.218 ± 62.581 | 306.322 ± 66.527 | 292.443 ± 84.083 |
| Hematology |  |  |  |  |  |  |
| Basophils (proportion of 1) | 0.005 ± 0.003 | 0.005 ± 0.002 | 0.006 ± 0.003 | 0.006 ± 0.003 | 0.007 ± 0.003 | 0.006 ± 0.002 |
| Eosinophils (proportion of 1) | 0.031 ± 0.024 | 0.030 ± 0.030 | 0.030 ± 0.025 | 0.034 ± 0.024 | 0.036 ± 0.026 | 0.029 ± 0.024 |
| Ery. Mean Corpuscular Hemoglobin (pg/cell) | 31.533 ± 2.426 | 31.450 ± 2.314 | 31.550 ± 2.430 | 31.667 ± 2.626 | 31.683 ± 2.384 | 31.400 ± 2.537 |
| Ery. Mean Corpuscular Volume (fL) | 94.100 ± 6.737 | 93.983 ± 6.905 | 93.817 ± 6.986 | 94.050 ± 7.081 | 94.017 ± 6.506 | 94.367 ± 7.050 |
| Erythrocytes (10E12/L) | 4.448 ± 0.403 | 4.343 ± 0.469 | 4.327 ± 0.446 | 4.188 ± 0.348 | 4.117 ± 0.450 | 4.413 ± 0.480 |
| Hematocrit (proportion of 1) | 0.418 ± 0.031 | 0.407 ± 0.034 | 0.404 ± 0.032 | 0.393 ± 0.031 | 0.385 ± 0.032 | 0.415 ± 0.030 |
| Hemoglobin (g/L) | 139.833 ± 10.534 | 136.000 ± 10.954 | 135.667 ± 10.152 | 132.167 ± 11.600 | 129.833 ± 10.439 | 138.000 ± 9.487 |
| Leukocytes (10E9/L) | 5.983 ± 0.993 | 6.083 ± 1.264 | 5.567 ± 0.628 | 5.550 ± 1.375 | 5.183 ± 0.884 | 5.550 ± 1.353 |
| Lymphocytes (proportion of 1) | 0.239 ± 0.085 | 0.216 ± 0.087 | 0.225 ± 0.054 | 0.234 ± 0.051 | 0.246 ± 0.058 | 0.229 ± 0.078 |
| Monocytes (proportion of 1) | 0.068 ± 0.015 | 0.070 ± 0.022 | 0.073 ± 0.016 | 0.093 ± 0.014 | 0.084 ± 0.016 | 0.072 ± 0.021 |
| Neutrophils (proportion of 1) | 0.675 ± 0.090 | 0.704 ± 0.116 | 0.667 ± 0.076 | 0.631 ± 0.055 | 0.627 ± 0.047 | 0.674 ± 0.089 |
| Platelets (10E9/L) | 239.167 ± 60.018 | 237.000 ± 51.198 | 233.167 ± 52.874 | 236.667 ± 45.562 | 230.500 ± 45.324 | 266.500 ± 49.529 |
| Urinalysis |  |  |  |  |  |  |
| Alpha-1 microglobulin (mg/L) | 4.917 ± 4.020 | 4.733 ± 6.186 | 5.083 ± 4.087 | 5.750 ± 6.291 | 8.167 ± 9.326 | 4.767 ± 3.942 |
| Urinalysis albumin (mg/L) | 27.167 ± 39.736 | 15.000 ± 16.852 | 16.500 ± 17.513 | 20.500 ± 26.516 | 30.833 ± 45.221 | 22.333 ± 23.560 |
| Urinalysis creatinine (umol/L) | 10422.213± 6111.824 | 8702.243 ± 5427.728 | 11281.755 ± 6361.686 | 8943.723 ± 3926.278 | 11080.793 ± 4843.667 | 9134.372 ± 3756.689 |
| Urinalysis erythrocytines (/HPF) | 1.000 ± 0.000 | 1.000 ± 0.000 | 1.000 ± 0.000 | 1.000 ± 0.000 | 1.000 ± 0.000 | 10.800 ± 21.913 |
| Urinalysis leukocytes (/HPF) | 1.000 ± 0.000 | 1.000 ± 0.000 | 1.000 ± 0.000 | 1.000 ± 0.000 | 1.000 ± 0.000 | 1.000 ± 0.000 |
| pH | 6.333 ± 0.258 | 6.500 ± 0.632 | 6.000 ± 0.316 | 6.083 ± 0.492 | 6.333 ± 0.408 | 6.083 ± 0.585 |
| Activated partial thromboplastin time (s) | 30.600 ± 1.126 | 31.100 ± 0.860 | 30.900 ± 1.437 | 30.950 ± 0.994 | 30.950 ± 0.994 | 30.976 ± 1.445 |
| Prothrombin intl. normalized ratio | 1.037 ± 0.041 | 1.058 ± 0.048 | 1.048 ± 0.053 | 1.025 ± 0.062 | 1.027 ± 0.030 | 1.012 ± 0.043 |
| Vital signs |  |  |  |  |  |  |
| Pulse rate (beats/min) | 60.00 ± 10.973 | 66.00 ± 11.730 | 65.83 ± 9.663 | 66.17 ± 9.131 | 64.00 ± 6.986 | 62.50 ± 9.524 |
| Systolic blood pressure (mmHg) | 134.67 ± 22.295 | 130.17 ± 21.442 | 121.17 ± 16.142 | 142.50 ± 17.501 | 125.33 ± 20.206 | 132.83 ± 27.096 |
| Diastolic blood pressure (mmHg) | 80.17 ± 7.055 | 81.33 ± 11.483 | 77.83 ± 10.926 | 79.83 ± 11.531 | 79.33 ± 9.668 | 81.67 ± 12.258 |
| Temperature (C) | 36.12 ± 0.172 | 36.03 ± 0.356 | 35.95 ± 0.288 | 36.07 ± 0.468 | 36.05 ± 0.451 | 36.07 ± 0.350 |
